# Brain Stroke Classification Using CT Scans with Transformer-Based Models and Explainable AI

**DOI:** 10.3390/diagnostics15192486

**Published:** 2025-09-29

**Authors:** Shomukh Qari, Maha A. Thafar

**Affiliations:** Department of Computer Science, College of Computers and Information Technology, Taif University, Taif 21944, Saudi Arabia; shmukh14200@gmail.com

**Keywords:** hemorrhagic stroke, ischemic stroke, stroke classification, deep learning, transfer learning, vision transformer, neuroimaging, explainable AI

## Abstract

**Background & Objective:** Stroke remains a leading cause of mortality and long-term disability worldwide, demanding rapid and accurate diagnosis to improve patient outcomes. Computed tomography (CT) scans are widely used in emergency settings due to their speed, availability, and cost-effectiveness. This study proposes an artificial intelligence (AI)-based framework for multiclass stroke classification (ischemic, hemorrhagic, and no stroke) using CT scan images from the Ministry of Health of the Republic of Turkey. **Methods:** We adopted MaxViT, a state-of-the-art Vision Transformer (ViT)-based architecture, as the primary deep learning model for stroke classification. Additional transformer variants, including Vision Transformer (ViT), Transformer-in-Transformer (TNT), and ConvNeXt, were evaluated for comparison. To improve model generalization and handle class imbalance, classical data augmentation techniques were applied. Furthermore, explainable AI (XAI) was integrated using Grad-CAM++ to provide visual insights into model decisions. **Results:** The MaxViT model with augmentation achieved the highest performance, reaching an accuracy and F1-score of 98.00%, outperforming the baseline Vision Transformer and other evaluated models. Grad-CAM++ visualizations confirmed that the proposed framework effectively identified stroke-related regions, enhancing transparency and clinical trust. **Conclusions:** This research contributes to the development of a trustworthy AI-assisted diagnostic tool for stroke, facilitating its integration into clinical practice and improving access to timely and optimal stroke diagnosis in emergency departments.

## 1. Introduction

Stroke is a major global health burden and a leading cause of death and long-term disability. According to the World Health Organization (2021), stroke accounts for approximately 10% of annual global deaths, totaling around 6.6 million fatalities each year [1]. There are various causes of stroke, including atherosclerosis, high blood pressure, and lifestyle factors such as lack of exercise, smoking, and alcohol consumption. Strokes are generally divided into two types: ischemic and hemorrhagic. Ischemic stroke is the most prevalent type, representing approximately 87% of all stroke cases [1]. It occurs due to a lack of blood flow to the brain caused by a blockage in the major blood vessels that a blood clot may block. This blockage leads to a lack of oxygen and nutrients in the brain, and consequently, the brain cells and tissues begin to die. The second type is Hemorrhagic stroke, which accounts for only 13% of all strokes. It happens when blood vessels that supply oxygen to the brain cells and tissues rupture and bleed, causing an increased pressure on the surrounding tissue inside the skull, which can further lead to brain damage.

Early stroke detection is the first essential step in stroke management, and it is crucial in improving survival rates, which exceed 90% [2]. Several imaging techniques have been utilized for stroke detection and diagnosis. Computed tomography (CT) and magnetic resonance imaging (MRI) are the two primary imaging modalities for stroke detection and classification, each offering unique advantages aligned with specific clinical requirements. The time factor is sensitive in stroke detection. Efficient, accurate, and fast stroke identification and determination of its type are critical to improve the patient’s outcome and to decide the treatment plan. Misclassification of the stroke type causes incorrect treatment since ischemic stroke requires treatments to restore blood flow for vessel blockage, while hemorrhagic stroke requires actions to control and stop bleeding [3]. Therefore, misclassification can lead to harmful consequences and cause death.

Stroke symptoms frequently appear suddenly and may be so subtle that they are easily missed. Once symptoms become evident, timely intervention becomes critical, as delays can significantly impact patient outcomes. Therefore, rapid clinical assessment and immediate access to diagnostic results are essential for effective stroke management. To support early detection, brain imaging modalities (i.e., MRI and CT scans) are commonly used [4]. While both play valuable roles, CT scans remain the preferred standard in emergency settings due to their speed, cost-effectiveness, and widespread availability in hospitals and clinics. Consequently, there is an urgent need for automated and reliable stroke detection systems that can assist clinicians in providing timely and accurate assessments.

In this study, we addressed the problem of stroke detection from two key perspectives. The first involves the development of an automated, AI-driven system that leverages CT scan imaging and integrates advanced AI techniques across various stages of a comprehensive pipeline. This system is designed to detect the presence of stroke and classify its type, either ischemic or hemorrhagic, in real-time, providing rapid support for clinical decision-making. The second perspective focuses on mitigating the challenges of limited and imbalanced training datasets as well as enhancing model interpretability. These include the limited size and diversity of available training datasets, which can impact model generalization, and the pressing need for interpretability in model predictions. In clinical practice, many healthcare professionals require a clear understanding of how AI systems reach their conclusions to build trust in the technology and support its adoption. To support this, we incorporated Explainable AI (XAI) techniques that provide visual interpretations of model predictions, improving transparency and clinical relevance.

To summarize, the contribution of this study folds into five main points:

1. Design of an end-to-end AI pipeline that integrates data preprocessing, augmentation, classification, and interpretability tailored for fast and accurate stroke detection.
2. Formulation of the problem as a multiclass classification task, enabling differentiation between ischemic, hemorrhagic, and normal cases from CT scan images.
3. Adoption and benchmarking of recent transformer-based and hybrid architectures, including MaxViT, TNT, and ConvNeXt, in the underexplored domain of CT-based stroke classification.
4. Utilization of GAN-based augmentation strategies to enhance model robustness and generalization.
5. Integration of Grad-CAM++ based Explainable AI, allowing for transparent, interpretable decision support that aligns with clinical expectations.

These contributions collectively advance the field by offering both methodological innovation and practical utility in clinical stroke diagnosis.

The remainder of this research is structured as follows: Section 2 provides background by explaining the medical-related and AI-related terms and concepts. Section 3 summarizes the relevant research literature and outlines the ML, DL, and transfer learning (TL) methods developed for stroke detection. Section 4 describes the dataset and methodology. Section 5 explains the experimental design, covering the evaluation metrics, the training/testing procedures, and the implementation details. Section 6 discusses the results and key findings. Finally, Section 7 concludes the study and suggests directions for future research.

## 2. Background and Concepts

Designing an AI model capable of accurately detecting and classifying stroke types requires a solid understanding of both the medical features of stroke and the imaging characteristics that can be leveraged by DL models. This section introduces essential concepts from the medical imaging perspective to inform how stroke patterns manifest in CT scans and how these patterns guide the training and interpretation of DL-based classification systems. The medical imaging concepts relevant to stroke detection are described as follows.

### 2.1. Stroke Overview

Stroke is a critical neurological condition that requires rapid detection and classification to ensure timely treatment. It is broadly categorized into two types.

Ischemic Stroke represents the largest part (approximately 87% of cases): This type results from an obstruction in cerebral blood vessels, typically due to intracranial thrombosis or extracranial embolism originating from the heart or major arteries [5]. The resulting lack of oxygen causes infarction, a region of dead brain tissue, which appears on CT scans as hypodense (low intensity) areas. The size of the infarct often correlates with the severity and duration of ischemia.

Hemorrhagic Stroke (around 13% of cases): Caused by the rupture of a blood vessel within or around the brain, leading to intracerebral hemorrhage (ICH) or subarachnoid hemorrhage (SAH) [6]. This bleeding forms a hematoma, visible on CT scans as hyperdense (bright) regions due to the pooling of blood. Hemorrhagic strokes are often linked to hypertension and can rapidly elevate intracranial pressure, causing significant damage.

### 2.2. Key CT Imaging Features for Stroke Classification

CT scans serve as the primary imaging modality in emergency stroke diagnosis due to their speed and accessibility. Distinguishing between ischemic and hemorrhagic strokes on CT relies on characteristic visual features:

- Ischemic strokes typically present as dark regions (i.e., hypodense), reflecting infarcted tissue where the blood supply has been blocked. The extent of hypodensity correlates with infarct size and stroke progression [7].
- Hemorrhagic strokes are indicated by bright regions (i.e., hyperdense) caused by acute bleeding. The shape, intensity, and location of these hyperdense areas help differentiate between ICH and SAH.

These imaging patterns, hypodense infarctions and hyperdense bleedings, are essential visual markers that deep learning models can learn to distinguish. Table 1 summarizes these and other relevant imaging features extracted from CT scans, which serve as critical inputs for pretrained models to automate stroke classification effectively [8].

## 3. Related Works

Over the past years, stroke has contributed significantly to global mortality rates. Traditionally, diagnosis relied on clinical assessments, neuroimaging techniques such as CT and MRI, and laboratory tests. Recently, there has been a notable shift toward integrating artificial intelligence (AI), more specifically machine learning and deep learning, into healthcare systems [9,10,11,12]. One of its primary applications is in the early detection of stroke, utilizing CT and MRI scans in combination with DL and ML techniques [13,14]. Given the limitations of current stroke treatment, early diagnosis plays a critical role in improving patient outcomes. AI-driven methods have shown promise in reducing diagnostic delays and facilitating timely interventions.

This section reviews recent studies (2020–present) focused on stroke detection, organized into three methodological categories: Machine Learning, Deep Learning, and Transfer Learning. These categories highlight major developments and emerging trends in stroke diagnosis.

### 3.1. Stroke Detection Techniques Using Machine Learning

Several studies utilized ML for stroke detection [4,15]. A comprehensive review by Sirsat and coauthors [4] examined 39 studies, categorizing ML-based approaches into four functional groups: stroke prevention, diagnosis, treatment, and prognostication. Among these categories, the diagnosis-focused studies are most aligned with the scope of this work, as they frequently employed CT scans for stroke identification. Their findings indicated that support vector machine (SVM) was the most effective classifier in ten studies, with SVM and Random Forest consistently delivering strong results across all categories.

Another notable study [16] presented a new approach to the classification of stroke subtypes, specifically hemorrhagic and ischemic strokes, using SVM, Multi-Layer Perceptron (MLP), Minimal Learning Machine (MLM), Linear Discriminant Analysis (LDA), and Structural Co- Occurrence Matrix (SCM). SCM is the best method to extract the most discriminant structural information concerning stroke subtype without requiring parameters tuning, achieved a classification accuracy of 98% without requiring hyperparameter tuning, using a dataset of 300 CT scans.

Despite the promising results reported in several studies that utilized ML for stroke identification, the ML approaches still have limitations. For example, ML heavily depends on manual feature engineering (i.e., extraction and selection), which often requires domain expertise, and it is a time-consuming process. Moreover, Traditional ML models often struggle to scale with large and complex datasets, especially when investigating high-dimensional CT images. These limitations justify the transition to DL models.

### 3.2. Deep Learning Approaches for Stroke Detection

Recent studies have explored early stroke detection using the DL techniques [17,18]. One of these studies [18] focused on the prediction of hemorrhagic stroke using CT scans obtained from the 2019-RSNA Brain CT Hemorrhage Challenge. The data was gathered from three institutions: Stanford University, Universidade Federal de São Paulo Brazil, and Thomas Jefferson University Hospital Philadelphia, and subsequently re-annotated by the American Society of Neuroradiology with the assistance of over 60 neuroradiologists [19]. The authors designed a DL approach that mimics the interpretation process of radiologists by combining a 2D Convolutional neural network (CNN) model and two sequence models to achieve accurate detection and subtype classification. The model achieved high prediction performances based on the area under the curve (AUC): intracranial hemorrhage (ICH): 0.988, epidural hematoma (EDH): 0.984, intraparenchymal hemorrhage (IPH): 0.992, intraventricular hemorrhage (IVH): 0.996, subarachnoid hemorrhage (SAH): 0.985, and subdural hematoma (SDH): 0.983.

Another study [20] introduced a new 13-layer CNN architecture named P-CNN for classifying CT scans into hemorrhagic, ischemic, and normal categories. Their model was found to be better than the well-known traditional CNN architectures such as AlexNet and ResNet50. P_CNN consists of input layer, convolution layer followed by a rectified linear unit (ReLU) activation function, Max pooling, and Dropout layers to prevent the overfitting, and a fully connected SoftMax layer. The classification accuracy obtained by their method in the first experiment is 98.33% and in the second experiment is 98.77%.

Similarly the study in [21] developed an automated approach to detect and quantitate infarction by using non–contrast-enhanced CT scans in patients with acute ischemic stroke. A ML approach to segmentation of infarction areas in non-optimized CT images in acute ischemic stroke patients showed good compatibility with stroke size on water-based MRI images.

Another recent study [22] focused on CNN-based architecture models developed a customized CNN model trained on 2501 CT images (1551 normal and 950 stroke) with an external multi-center test set of 9900 images. The model architecture, comprising three convolutional blocks and dense layers, was optimized through data augmentation and hyperparameter tuning. It achieved a validation accuracy of 97.2% and a recall of 96%. Notably, interpretability tools such as LIME, saliency maps, and occlusion sensitivity were integrated, enhancing clinical trust and demonstrating readiness for real-time deployment.

While DL-based methods have enhanced stroke detection and classification by automating feature extraction with high prediction accuracy, they still face some challenges, such as the need for a massive dataset to train the model from scratch, which is very time-consuming and has high computational costs. Therefore, TL has emerged to eliminate some of these issues.

### 3.3. Transfer Learning with Pretrained Models (CNN/Transformer)

Transfer learning (TL) has emerged as a practical solution to overcome several limitations associated with DL approaches, particularly by leveraging pretrained models tailored for vision tasks in the medical imaging domain. Notably, Vision Transformer (ViT)-based architectures have been increasingly adopted in disease diagnosis from medical images, demonstrating promising results across various applications [23,24,25,26]. Recent studies that utilized TL with pretrained models to advance stroke detection and classification tasks can be categorized to CNN-based and Transformer-based architectures. One of these studies [27] developed a novel CNN architecture for detecting and classifying brain stroke into two classes (hemorrhagic and ischemic) and three classes (hemorrhagic, ischemic, and normal) using non-contrast brain CT images. CNN and U-Net were employed to classify strokes and normal, and then image segmentation was utilized to detect the stroke type. The model’s architecture consists of 19 layers, and the model achieved a 95.06% for the classification model. However, this study relied on a private dataset, limiting reproducibility and external benchmarking. In contrast, another study [28] demonstrates the effectiveness of TL by utilizing pretrained CNN models for stroke classification from CT images. The study employed well-established CNN architectures, including EfficientNet-B0, ResNet50, and VGG19, which were pretrained on large-scale datasets such as ImageNet dataset. These pretrained models were used to extract high-level features from brain CT scans. After that, these features were fed to traditional ML algorithms such as SVM and K-Nearest Neighbors (KNN) for classification into three classes (ischemic stroke, hemorrhagic stroke, and normal brain). By combining DL/TL-based feature extraction with ML classifiers, this hybrid approach achieved exceptional results, with the EfficientNet-B0 + SVM model achieving the highest accuracy of 95.13%, recall of 94.93%, precision of 95.06%, and an F1-score of 94.94%. Notably, this study used a dataset provided by the Republic of Turkey’s Ministry of Health, collected via the e-Pulse and Teleradiology System. Although this dataset is not publicly available, it was obtained with institutional permission, allowing for reproducibility within approved research contexts.

Another study [29] utilized state-of-the-art vision transformers (ViT Base 16) for stroke classification. The study employed the pretrained VIT model to classify CT scan images into three categories: Not stroke, Hemorrhagic stroke, and Ischemic stroke. The ViT model, which leverages the power of self-attention mechanisms, was fine-tuned to adapt to the stroke classification task, demonstrating its effectiveness. The experiment showed an accuracy score of 91% for the best classification model developed in this study. Although this study highlights the advantages of transformer-based pretrained models for medical image classification, mainly for tasks of stroke classification using CT scans, some advanced transformer-based pretrained models with effective capability to capture both local and global features have not been explored in addressing such a problem.

In the same direction, [30] explored transfer learning with a Vision Transformer (ViT-B16) model for acute ischemic stroke identification from MRI scans. They fine-tuned a ViT-B16 (pretrained on ImageNet) on a 342 T1-weighted MRI images (stroke vs. normal cases from Moroccan hospitals) dataset. The MRI data underwent standardization and image augmentation (random flips and zoom transformations) to improve generalization. The ViT model’s transformer encoder was combined with new dense layers and trained for 50 epochs, with optimization via Adam. This ViT-based model achieved an impressive accuracy of 97.6% in classifying ischemic stroke, substantially higher than a conventional VGG16 CNN baseline (90% accuracy) on the same MRI set. ViT-B16 outperformed multiple CNN benchmarks (e.g., ResNet50 at 87%, InceptionV3 at 82%) from a prior study. The model also demonstrated high precision and recall (96–98% for both classes). These results illustrate that transformer-based transfer learning can improve stroke detection accuracy over traditional CNN methods, underlining the potential of advanced pretrained models in medical imaging.

Additionally, a very recent study [31] focused on hemorrhagic stroke (intracranial hemorrhage) detection in CT scans using transfer learning. The study leveraged the large RSNA intracranial hemorrhage CT dataset, fine-tuning a pretrained VGG-16 model (after considering other CNN backbones like AlexNet, EfficientNet-B2, ResNet50, MobileNet, and Inception) to classify hemorrhages into five subtypes plus normal. The VGG-16’s final classification layer was modified to output six classes, and data augmentation techniques (e.g., rotations and contrast enhancement) were applied to improve robustness during fine-tuning. Despite the limited training data per class, the model achieved high performance—about 86.5% overall accuracy, with ~85.9% precision, ~86.2% recall, and ~86.7% F1-score on hemorrhage classification. This demonstrates robust transfer learning performance for hemorrhage detection on CT. The author noted some generalizability challenges (due to scanner and protocol variability) and plans to incorporate further enhancements, such as improved contrast.

Another recent study [32] evaluated pretrained CNNs (Xception, VGG16, VGG19, InceptionV3, InceptionResNetV2) for stroke detection in CT scans. Transfer learning followed by fine-tuning significantly boosted accuracy, VGG16 and VGG19 reached 97.2%, up from 92% with standard TL. These results underscore the power of model adaptation in improving diagnostic performance while reducing system burden.

Despite the success that has been made toward stroke detection and classification, there are still some limitations or gaps that can be addressed for future studies. For example, although DL models have been developed to detect and classify strokes, the prediction performance is still limited. Furthermore, current models often fail to be generalized well when dealing with new and unseen datasets due to changes in the quality of CT images and different imaging protocols. Another limitation, most of the existing studies use CNNs to detect strokes using CT scans, with a few efforts to explore transformer-based models. Also, the lack of use of data enhancement techniques where advanced data augmentation techniques are still underused in stroke detection, which can overcome the issue of limited medical dataset size and diversity. The last gap to highlight is the insufficient focus on the results interpretability of advanced AI models.

## 4. Materials and Methods

### 4.1. The Proposed System Workflow

**Figure 1:**
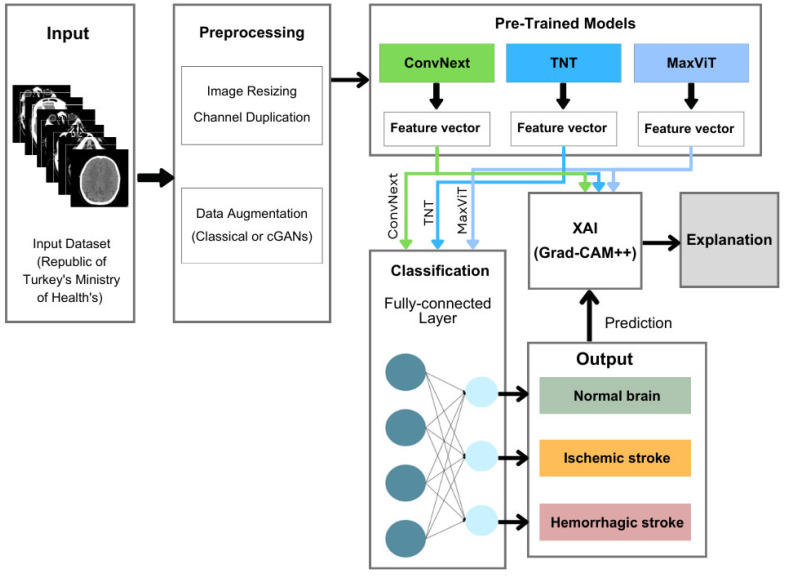
Overview of the proposed stroke classification pipeline. CT images are preprocessed and passed through transformer-based models (ConvNeXt, TNT, MaxViT) to extract features. Predictions are made via a fully connected layer, and interpretability is enhanced using Grad-CAM++ for clinical explanation.

In this study, stroke identification is represented as supervised learning of multiclass classification. The datasets consist of CT-scan images, and the goal is to predict if the image is normal, ischemic stroke, or hemorrhagic stroke. The methodological framework, illustrated in Figure 1, consists of the following main steps as follows:

1. Data Acquisition: to obtain the CT scans of the three classes.
2. Data Preprocessing and augmentation: The data is preprocessed to enhance image quality, and then data augmentation is applied using classical and GAN-based techniques.
3. Transfer Learning of Pretrained Models: the processed CT scan images are then used as inputs for the DL pretrained models to extract discriminative features automatically. These pretrained models are mainly ViT, MaxViT, ConvNext, and TNT.
4. Classification: Those auto-extracted features are fed into classification blocks using fully connected layers and a SoftMax function to produce an output in one of the defined classes (No Stroke, Hemorrhagic Stroke, or Ischemic Stroke).
5. Explainable AI: XAI clarifies the classification output, ensuring that the results are clear and convincing for physicians.

Each component of this pipeline is elaborated in detail in the following Section 4.2, Section 4.3, Section 4.4, Section 4.5 and Section 4.6, where we described the dataset sources, preprocessing operations, augmentation techniques, pretrained model architectures, classification procedures, and explainability strategies.

### 4.2. Datasets

The CTscan images dataset utilized in this study are obtained from the dataset published in a recent study for brain stroke detection [28]. It originates from the Republic of Turkey’s Ministry of Health, collected via the e-Pulse and Teleradiology systems during 2019–2020. The dataset includes anonymized CT brain images in both DICOM and PNG formats to ensure patient privacy in compliance with ethical standards. Image annotations were conducted by seven radiologists and subsequently validated by another specialist to guarantee clinical accuracy. Importantly, the dataset contains only imaging data and does not include any demographic or acquisition metadata, which could have added further clinical context but were intentionally excluded for privacy protection. Despite this, the dataset remains a valuable resource for developing and benchmarking deep learning models in stroke classification.

For this study, we utilized the original PNG-format images available in the dataset, as they are well-suited and more directly compatible with DL pipelines without the need for DICOM decoding or preprocessing. The dataset contains three classes: Ischemic stroke, Hemorrhagic stroke, and no stroke (normal brain scans). Table 2 provides a statistical summary of the dataset utilized in our experiments. As shown in the table, the classes of stroke are imbalanced, with the hemorrhagic and ischemic stroke categories having notably fewer samples compared to the normal class. Thus, to mitigate the impact of the imbalance issue, we applied advanced data augmentation techniques (i.e., GAN) on minor classes, “Hemorrhagic and Ischemic stroke,”. Additionally, a weighted loss function was applied during training to further address class imbalance. Further details of data augmentation are discussed in Section 4.3.

### 4.3. Data Preprocessing

This study utilizes CT-scan images for training three state-of-the-art DL pretrained models: TNT, MaxViT, and ConvNext. To ensure compatibility and optimal performance across these architectures, careful preprocessing was performed. All images were resized to a fixed resolution of 224 × 224 pixels, consistent with the input size of models pretrained on ImageNet dataset. Additionally, since CT images are grayscale (single-channel) but pretrained models require three-channel (RGB) input, the grayscale channel was replicated across all three channels to meet the input specifications while avoiding color bias. These standardized preprocessing steps ensure consistency, reduce computational variation, and enable fair evaluation across all models.

### 4.4. Data Augmentation

Data augmentation was employed to overcome class imbalance, increase dataset diversity, and improve the generalizability and robustness of the models. Two augmentation strategies were applied: classical augmentation techniques and conditional generative adversarial network (cGAN) technique. All augmented data, whether generated using classical techniques or cGAN, were used exclusively in the training set. These images were not included in the validation or test sets to preserve evaluation integrity and ensure fair model assessment.

#### 4.4.1. Classical Augmentation

We applied several standard image augmentation techniques to enrich the diversity of the training data, improve generalization, and prevent overfitting. These include geometric transformations such as random cropping, horizontal flipping, and rotation, as well as photometric transformations like color jittering. These transformations were applied dynamically during training, enriching the model’s exposure to varied representations of each class.

#### 4.4.2. Conditional GAN-Based Augmentation

Conditional Generative Adversarial Network (cGAN) is an extension of the traditional GAN model, with conditional information added to both the generator and the discriminator, allowing the model to generate images based on class labels [33]. It generates CT-scan images, specifically for minor classes (i.e., hemorrhagic and ischemic). These images are synthetic, but they mimic the original data. The cGAN framework comprises two adversarial components:

- The generator creates synthetic (i.e., fake) images based on random noise vectors and the class label.
- The discriminator evaluates the generated image by distinguishing between real and synthetic images.

Through adversarial training, the generator progressively improves its output quality based on feedback from the discriminator [34]. As a result, high-quality, class-specific synthetic images were produced and integrated exclusively into the training set to improve class balance and enhance the model’s ability to recognize underrepresented stroke types.

### 4.5. Pretrained Model Selection and Feature Extraction

To classify stroke types from brain CT scans, we employed state-of-the-art pretrained models that efficiently extract discriminative features. These models were originally trained on large-scale datasets such as ImageNet [35], have demonstrated an outstanding capability in capturing low-level patterns (e.g., edges and textures) and high-level semantic representations (e.g., global context). We adapted these models to the medical imaging domain in a transfer learning style. The selected models fall into two main categories: First, Transformer-based architectures: The Vision Transformer (ViT) and Transformer-in-Transformer (TNT). Second, Hybrid convolution-transformer architectures: ConvNeXt and MaxViT). Among these models,

- ViT serves as the baseline model for this study, providing a reference point for evaluating the improvements introduced by more advanced designs.
- TNT is included for its hierarchical dual-transformer structure, which models both intra-patch and inter-patch relationships,
- ConvNeXt bridges traditional CNNs and transformers, integrating convolutional inductive biases with transformer-inspired components.
- MaxViT serves as the core model in our study, integrates multi-axis attention with hierarchical design. It stands out as a powerful architecture for medical image analysis.

Below, we briefly describe the architecture and motivation behind each model.

(1) ***Vision Transformer (ViT) Pretrained Model***

The Vision Transformer (ViT) [36], inspired by the success of transformers in NLP, was developed specifically for computer vision tasks. ViT is the foundation of transformer-based architectures successfully applied to image classification. This approach first divides an input image into multiple non-overlapping patches. Each patch is flattened and converted into a vector, followed by dimensionality reduction through a linear projection. A transformer encoder subsequently processes these vector embeddings. This allows the ViT to capture local and global patterns in the image, which is crucial for analyzing CT scans where stroke-related features may be subtle and widely distributed.

(2) ***Transformer-in-Transformer Pretrained Model***

The Transformer-in-Transformer (TNT) [37] model, specifically the tnt_s_patch16_224 variant from the Timm library, extends ViT by introducing a nested attention structure. TNT enhances the feature representation ability by dividing each input image into several patches as “visual sentences” and further subdividing each patch into sub-patches as “visual words”. The TNT architecture consists of two core components:

- Inner transformer, which models the relationship between visual words for local feature extraction (local representations).
- Outer transformer, which captures the intrinsic information from the sequence of sentences (global representations).

**Figure 2:**
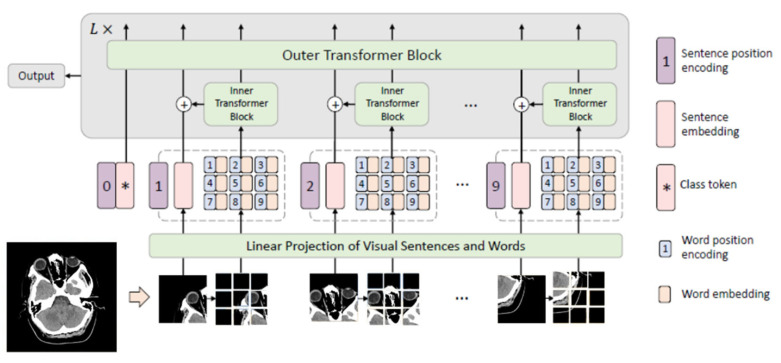
Illustration of the Transformer-in-Transformer (TNT) Architecture [37] showing the nested attention mechanism with inner and outer transformer blocks. The input CT image is tokenized into visual sentences and visual words to capture both local and global features. This design supports rich representation learning for medical image analysis.

As illustrated in Figure 2, this dual-level attention mechanism enhances representational capacity for both local stroke signs (e.g., subtle infarcts) and global context (e.g., midline shifts or edema). Each component in TNT architecture plays a crucial role in transforming raw CT-scan images into meaningful features for stroke classification.

(3) ***Convolutional Neural Network Next (ConvNeXt) Pretrained Model***

ConvNeXt [38] modernizes traditional CNNs by incorporating design elements from transformers. We used the convnext_base variant from the Timm library due to its strong performance in various medical imaging tasks. These improvements of classic CNN include replacing the batch normalization with layer normalization and increasing the convolutional kernel size (from 3 × 3 to 7 × 7) to approximate the global self-attention mechanism in transformers. Key improvements include:

- Larger convolution kernels (7 × 7) to simulate global receptive fields.
- Layer normalization and GELU activations for improved convergence.
- Effective at detecting hyperdense (hemorrhagic) and hypodense (ischemic) regions in CT scans.

This architecture offers a balance between efficiency and accuracy, especially suitable for grayscale radiology images.

(4) ***MaxViT Hybrid Pretrained Model***

The Multi-Axis Vision Transformer (MaxViT) model [39], also implemented via Timm library, is a hybrid transformer that leverages two attention mechanisms: local and global attention within the transformer framework while also incorporating CNN-like features into its architecture. MaxViT’s core innovation lies in its multi-axis attention mechanism, which integrates two complementary attention types as follows:

- Grid Attention: which focuses on small, localized patches to capture fine details, such as infarcts or localized hemorrhage, and
- Axial Attention: which looks along the horizontal and vertical directions to understand the overall structure of the image.

MaxViT follows a hierarchical CNN-like design while embedding transformer-based attention mechanisms across multiple stages. We selected the MaxViT model for several reasons:

1. It balances extracting subtle local features with understanding the global context. In the context of stroke detection, the features are categorized as local features that help detect subtle changes in brain structures that indicate strokes, and global context that ensures consideration of the broader anatomical structure, which is crucial for precisely classifying stroke types.
2. Built-in segmentation capability eliminating the need for external segmentation models like UNet. MaxViT inherently segments feature during classification.

### 4.6. Transfer Learning, Fine-Tuning, and Classification

To leverage existing knowledge embedded in large-scale pretrained models, we adopted a transfer learning approach tailored for stroke classification using brain CT scans. As outlined in Section 4.5, the selected models: MaxViT, ConvNeXt, and TNT were originally trained on the ImageNet dataset [35,40], and are known for their ability to capture both low-level visual cues and high-level semantic information. By applying TL, the pretrained models are fine-tuned to adapt their learned features to the domain-specific task, which is stroke classification. This process ensures vital feature representation and significantly reduces the computational cost and training time compared to training models from scratch. It also improved the prediction accuracy for this application. To adapt the models to our clinical task, we fine-tuned each architecture through the following steps:

1. Classification Head Adaption: the original classification layers of each model were replaced with a fully connected layer consisting of three output neurons, corresponding to one of the predefined stroke categories: Class 0: No Stroke, Class 1: Hemorrhagic Stroke, or Class 2: Ischemic Stroke.
2. SoftMax Activation for Multiclass Prediction: The final layer uses the SoftMax activation function to compute a probability distribution across the three classes, enabling multiclass predictions.
3. Layer Freezing and Fine-Tuning: For each model, all layers were frozen except for the final classification layer, to take advantage of the pretrained weights in the feature extraction stages, while allowing the classification layer to adapt to the characteristics of our specific clinical task using the new dataset.
4. Hyperparameter Configuration: The models were trained with a variety of hyperparameter settings. The most important parameters we have optimized include batch size, learning rate, the optimizer, and the number of epochs. These configurations were experimentally tuned to optimize each model’s performance on the CT stroke classification task. The finalized settings are summarized in Table 3.

By combining transfer learning with careful classifier design and tuning, we ensured that the models were effectively repurposed for stroke detection from CT scans, achieving high efficiency and predictive performance. suitable for clinical integration. Further implementation details regarding the packages and libraries of pretrained models, computational environment, and training setup are described in Section 5.4.

### 4.7. Explainable AI (XAI) Integration

To enhance the transparency and reliability of the stroke classification model, we integrated Explainable AI (XAI) techniques, specifically Gradient-weighted Class Activation Mapping (Grad-CAM). Grad-CAM generates visual explanations for CNNs by identifying and highlighting the most important regions in the input image that contribute significantly to the model predictions. Grad-CAM depends on calculating the gradients of the result of a class with respect to the feature maps of a convolutional layer to produce a heatmap highlighting the most important regions [41].

This visualization technique is particularly beneficial in the medical domain, where interpretability is critical. In clinical practice, understanding the model’s prediction process enables physicians to build trust in AI-assisted decisions. By localizing discriminative areas, such as infarcted or hemorrhagic regions, Grad-CAM assists clinicians verify whether the model’s focus aligns with radiological findings [42].

In this study, we used Grad-CAM++ [43], an advanced extension of Grad-CAM that provides better localization and sharper heatmaps, particularly effective when multiple object regions contribute to the decision. Grad-CAM++ enhances the model interpretability by refining the attribution of importance, making it more suitable for complex tasks like stroke subtype classification. We applied Grad-CAM++ to CT scan images to visualize and validate the decision-making process of our model.

## 5. Experiments and Evaluation Protocols

This section outlines the experimental design used to evaluate the performance of the proposed AI-based framework for stroke detection and classification. It includes the setup of the training and testing processes, dataset splitting strategies, and key evaluation metrics.

### 5.1. Evaluation Metrics

Evaluation metrics are an integral part of the model as they assess its performance and help verify its efficiency. To evaluate our models, we utilized *Accuracy*, *Precision*, *Recall*, *F*1-*score*, and the Area Under the ROC Curve (AUC) metrics. These metrics are derived from the confusion matrix, which consists of four key components: True Positive (*TP*), True Negative (*TN*), False Positive (*FP*), and False Negative (*FN*).

**Accuracy**: One of the most important metrics used in classification tasks. It represents the ratio of correctly predicted instances (*TP* and *TN*) to the total number of instances. *TP* and *TN* are correctly predicted, while *FP* and *FN* are incorrectly predicted. The formula for accuracy is as follows:

(1) $$Accuracy=\frac{\;TP+TN}{TP+TN+FP+FN}$$

**Precision**: This metric indicates how many predicted positive values are positive. The formula for precision is as follows:

(2) $$Precision=\frac{\;TP}{TP+FP}$$

**Recall**: *Recall* measures how well the model identifies true positive instances (e.g., detecting all actual stroke cases). The formula for recall is as follows:

(3) $$Recall=\frac{\;TP}{TP+FN}$$

**F1 Score**: This metric provides a harmonic mean of *Precision* and *Recall*, useful when a balance between them is important. The formula for the *F*1 *Score* is as follows:

(4) $$F1\;Score=2*(\frac{Precision\;*\;Recall}{Precision+Recall})$$

**Area Under the ROC Curve (AUC)**: Evaluates the model’s ability to distinguish between classes. It visualizes the trade-off between the True Positive Rate (Sensitivity) and the False Positive Rate (1 − Specificity) across all thresholds. AUC values close to 1.0 indicate strong class-separability.

These metrics were computed for each model variant (ViT, TNT, MaxViT, ConvNeXt), enabling performance comparison and selection of the best-performing architecture for clinical applicability.

### 5.2. Data Splitting Protocols

To ensure a consistency and fair comparison with baseline methods, we adopted a standard train-test splitting strategy. Moreover, to maintain the original class distribution and address potential class imbalance, the splitting was applied in a stratified style, where each class (No Stroke, Hemorrhagic Stroke, and Ischemic Stroke) is proportionally represented in both the training and testing sets. The data was split as follows:

- Training set: 80% of the data was used to train the models for classification.
- Testing set: 20% of the data was reserved to evaluate the performance of the final model on unseen test samples.

### 5.3. Data Augmentation Using cGAN Experiment

To address class imbalance issue, particularly the underrepresentation of Hemorrhagic and Ischemic stroke classes, we employed a conditional Generative Adversarial Network (cGAN) to synthesize realistic CT images.

The generator accepts a random noise vector along with a class label (Normal, Ischemic, or Hemorrhagic) and outputs a grayscale image. It consists of several Dense and Conv2DTranspose layers to reconstruct the visual structure of the image. The discriminator received both real and generated (i.e., synthetic) images with their associated class labels, distinguishing between real and fake samples using a set of Conv2D layers followed by Dense. During training, the generator and the discriminator are alternately updated using their respective loss functions. The generator learns to produce images capable of deceiving the discriminator, while the discriminator is trained to enhance its ability to detect fake images. The model was trained in two distinct phases:

- Phase 1 (Stabilization Phase): The cGAN was trained for 200 epochs, and the quality of the generated images stabilized noticeably. However, these images were not saved during this phase.
- Phase 2 (Generation Phase): With the image-saving mechanism activated, the training continued for an additional 800 epochs. At each epoch, approximately 800 synthetic images were generated to balance the dataset (split equally between Ischemic and Hemorrhagic classes). These images were automatically stored in class-specific folders.

**Figure 3:**
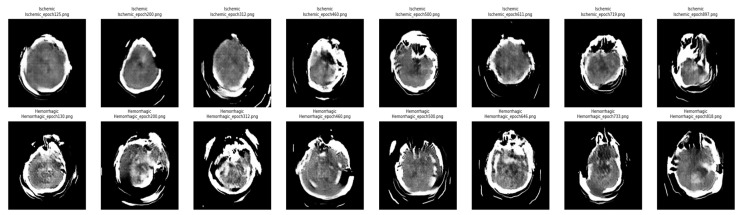
Progressive synthetic CT-scan images generated by the conditional GAN (cGAN) for the Ischemic (**top row**) and Hemorrhagic (**bottom row**) stroke classes. Images in the leftmost columns (e.g., Epochs 125–130) show lower visual quality during early training, whereas images in the rightmost columns (e.g., Epochs 719–897) demonstrate improved anatomical structure and quality.

After completing the training, the quality of the generated images was assessed, showing significant similarity to the original CT scans. Based on this assessment, synthetic images for the Hemorrhagic and Ischemic categories generated during Phase 2 were integrated with the original training data for these two categories. No synthetic images were added for the Normal category to preserve class balance. As previously noted in Section 4.4, these synthetic images were used exclusively for training and were not included in the validation or test sets to ensure a fair evaluation. Figure 3 illustrates a sequence of synthetic CT images generated using the cGAN framework for both Ischemic (top row) and Hemorrhagic (bottom row) stroke classes. The images are captured at various training epochs, allowing visual examination of the model’s progression in learning crucial stroke features. The synthetic images from earlier epochs (such as epochs 125 and 130) show blurred anatomical structures, which suggest early-stage fluctuation and mode failure problems. Image quality improved significantly in later epochs (after 500 for Ischemic, and 664 for Hemorrhagic). Overall, the qualitative advancement demonstrates that cGAN effectively captures modality-specific stroke features.

### 5.4. Implementation Details and Resources

We implemented our proposed method using a combination of local and cloud-based computational resources to support both model development and large-scale experimentation.

Initial development and testing were conducted on a local ASUS X415JF laptop equipped with an Intel Core i7 (10th generation) processor, NVIDIA GeForce MX330 GPU, 16 GB of RAM, and a 1 TB SSD. For large-scale training deep learning models and running extended experiments, we leveraged Google Colab Pro, which provides access to high-performance GPUs (Tesla T4 and P100) and extended memory capacity. This cloud environment was selected for its accessibility, scalability, and cost-effectiveness, enabling faster training and greater resource flexibility.

All pretrained models used in this study (e.g., ViT, TNT, MaxViT, ConvNeXt) were loaded from the Timm library (PyTorch Image Models), a reliable and popular resource that offers access to hundreds of cutting-edge vision models pre-trained on ImageNet. This library supports seamless integration and transfer learning functionalities, which are essential for our work. The Torchvision library was used for data processing and transformations, and scikit-learn for calculating performance metrics such as Precision, Recall, F1-score, and AUC. This combination of tools ensured robustness, reproducibility, and efficiency across all stages of the experimentation pipeline.

## 6. Results and Discussions

### 6.1. AI Model Prediction Performance

We present and discuss the prediction performance results obtained from four transformer-based and hybrid pretrained models used in this study. Each model was trained on 80% of the dataset and evaluated on the remaining 20% using standard classification metrics, including accuracy, precision, recall, F1-score, and AUC, as summarized in Table 4. The primary objective was to classify CT scans into one of three categories: hemorrhagic stroke, ischemic stroke, or normal (no stroke). To evaluate the impact of data augmentation, we designed three experimental setups per model: (1) training on the original dataset, (2) training with classical augmentation, and (3) training with synthetic images generated via cGAN.

As shown in Table 3, all models demonstrated strong predictive performance across the three experiments. Among them, MaxViT consistently achieved the best results, while ViT recorded the lowest across all metrics. This can be attributed to ViT relatively simple architecture, which lacks the hierarchical and multi-scale attention mechanisms present in more advanced models like TNT and MaxViT. These mechanisms help extract fine-grained features critical for medical image classification. In their baseline configurations (non-augmented), TNT, ConvNeXt, and MaxViT achieved competitive accuracies of 93.83%, 95.64%, and 97.67%, respectively. However, performance improved further with classical augmentation and showed even greater gains when using cGAN-generated synthetic data. Notably, cGAN augmentation had the strongest impact on TNT and ConvNeXt, each improving accuracy by nearly 2%. In contrast, MaxViT showed only a low improvement, increasing to 98.00%. This limited gain is likely due to MaxViT’s robust architecture, which combines block attention, grid attention, and convolutional layers to learn efficiently even from smaller datasets.

Moreover, to provide a more comprehensive evaluation, we additionally calculated the AUC metric, which reflects the model’s ability to distinguish between classes. As shown in Table 4, all models achieved high AUC and the scores consistently improved across all models when trained with augmented datasets either through classical augmentation or cGAN-based methods. The highest AUC was achieved by MaxViT (0.9975) when trained with cGAN augmentation. These results highlight the effectiveness of using conditional GANs in handling class imbalance, thereby enhancing the model’s discriminative capability. The consistently high AUC values confirm that the proposed models, particularly MaxViT, are well-suited for multiclass stroke classification with strong generalization and reliability.

In summary, MaxViT consistently outperformed all other model architectures across the three training setups: without augmentation, with classical data augmentation, and with cGAN-based augmentation. Among these, MaxViT combined with cGAN achieved the highest accuracy of 98.00%, followed closely by its other variants. When comparing the best-performing versions of each architecture, ConvNeXt with cGAN achieved the next highest accuracy at 97.45%, followed by TNT with cGAN at 96.00%. These findings highlight the superior generalization capability of MaxViT regardless of the augmentation strategy and further confirm the effectiveness of transformer-based architectures coupled with synthetic data augmentation for improving stroke classification performance.

### 6.2. Comparison with the State-of-the-Art Methods

To investigate our method’s prediction performance and illustrate its effectiveness, we conducted a comparative analysis against the baseline method introduced in the study titled *“Classification of brain ischemia and hemorrhagic stroke using a hybrid method”* [28], which remains the only publicly accessible work that performs multiclass stroke classification using the same dataset employed in our study. While other studies, such as [27], also address three-class classification tasks, their datasets are not publicly available, and thus could not be used for a direct and fair comparison under the same experimental conditions.

This baseline method employed a hybrid pipeline comprising EfficientNet-B0 as a feature extractor and SVM as a classifier. The dataset used in that study was collected from the Turkish Ministry of Health’s “e-Nabz” and “Remote Radiology” systems, with radiologist-labeled categories: ischemic, hemorrhagic, and normal. Preprocessing steps included resizing, contrast enhancement, denoising, and normalization. The hybrid model in the study relies on utilizing DL capabilities to extract representative features from the images and then passing these features to an SVM model to complete the classification.

To ensure a fair comparison, we adopted the same dataset, problem formulation, data splitting protocol, and evaluation metrics as the baseline study. Our evaluation focused on Accuracy, F1-score, Recall, and Precision. Table 5 shows the comparative results, which demonstrate that all three of our proposed advanced transformer-based and hybrid-based models consistently outperformed the baseline EfficientNet-B0 + SVM model across all evaluation metrics, with MaxViT achieving the highest performance, surpassing the baseline by 2.87% in Accuracy, 3.06% in F1-score, 3.07% in Recall, and 2.94% in Precision.

This performance gain underscores the advantage of using advanced attention-based architectures combined with synthetic data augmentation (cGAN), enabling more effective generalization and feature learning compared to traditional hybrid approaches.

### 6.3. Explainable AI Results and Insights

In high-stakes domains such as healthcare, deploying AI systems requires not only high classification performance but also interpretability and transparency. Therefore, we incorporated Explainable Artificial Intelligence (XAI) technique to clarify the features or areas within CT-scan images that the model relied upon during classification.

#### 6.3.1. Visual Interpretation Using Grad-CAM++

To visually interpret the classification decisions of the proposed MaxViT model, we applied the Grad-CAM++ a class-discriminative visualization technique. Grad-CAM++ generates class-specific heatmaps that highlight important regions within the input CT scans, reflecting the areas the model focused on when predicting stroke classes. We examined three different levels within the model to evaluate their ability to focus on relevant regions:

- Early Layer: captures broad low-level features.
- Mid-Level Layer: encodes structured intermediate representations.
- Deep Layer: captures high-level semantics related to stroke.

**Figure 4:**
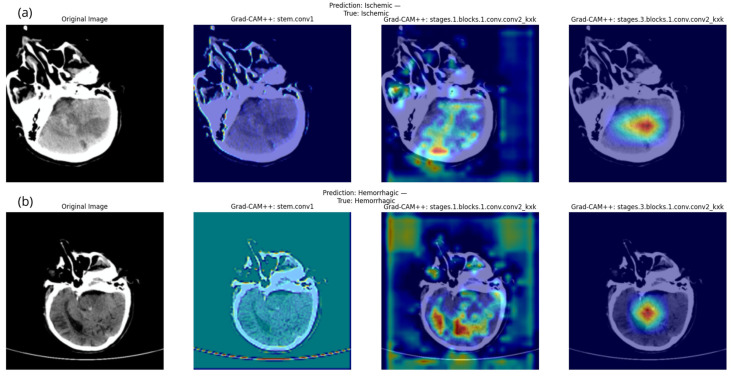
Grad-CAM++ visualizations across three convolutional layers for stroke classification using the MaxViT model. (**a**) Ischemic stroke case. (**b**) Hemorrhagic stroke case. For each case, the first column displays the original CT image, while the second, third, and fourth columns represent heatmaps from the early, mid-level, and deep layers, respectively. The deep layer consistently exhibits more focused and clinically aligned activation.

Figure 4a,b illustrates the Grad-CAM++ visualizations across these three layers for two representative cases: one ischemic (top) and one hemorrhagic (bottom). The first column shows the original input image. The second column displays the early layer’s activation that highlights broad and low-relevance regions, which does not show any actual focus on the important brain areas. The third column presents a mid-level layer with partially relevant activation but scattered focus. The fourth column corresponds to the deep layer, which accurately focuses on the core brain lesion areas related to correct classification, making it the most suitable for interpreting the model’s decisions. These findings support the deep layer’s effectiveness for interpretation purposes.

**Figure 5:**
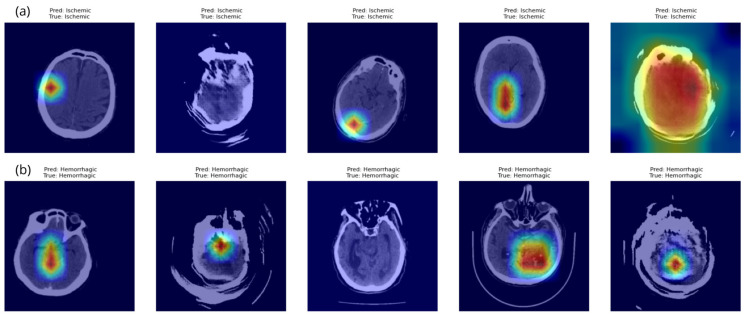
Grad-CAM++ visualizations for multiple correctly predicted stroke samples. (**a**) Ischemic stroke; (**b**) Hemorrhagic stroke. Each column shows the original CT image overlaid with the activation map from the model’s deep layer. Heatmap color intensity (from blue to red) represents the region’s contribution to the final classification.

In addition to layer-wise inspection, we provide Figure 5, which includes a broader selection of correctly predicted ischemic and hemorrhagic CT scans. This figure offers further insight into the model’s behavior across multiple examples of both stroke types, revealing that the model exhibits more localized and confident attention in hemorrhagic cases compared to ischemic ones, indicating differences in the model’s response to each type of image. To better understand the heatmaps on the CT scans and further interpret the visualizations from both Figure 4 and Figure 5, we examined the following key aspects:

- Color intensity: The color gradient, ranging from blue to red, indicates the degree to which a region contributed to the model’s prediction. The red color indicates the severity of importance in the model’s decision, as the darker the red color in a specific area, the more the model relies on that area in making its final decision. In contrast, the blue areas indicate little to no contribution.
- Shape and Localization: Heatmaps with focused dark red regions suggest confident and localized attention, likely corresponding to stroke-affected areas.
- Visual Alignment with Lesions: Although no separate segmentation mask technique was used, visual inspection indicates that the darkest red areas in the heatmaps often align with radiological signs of hemorrhage or ischemia. This overlap supports the validity of the model’s focus and adds confidence in its interpretability.

These insights reinforce the utility of Grad-CAM++ in validating and interpreting deep models for clinical decision-making in stroke detection.

#### 6.3.2. Quantitative Evaluation of Grad-CAM++ Explanations

To further evaluate the interpretability and localization performance of the Grad-CAM++ saliency maps generated from the MaxViT model, we conducted a quantitative analysis using three commonly adopted metrics: Area Under the ROC Curve (AUC), Dice Coefficient, and Average Activation (AvgActivation) and reported them in Table 6.

These metrics provide complementary insights into the effectiveness of model explanations in localizing stroke-relevant regions as follows:

- AUC (Area Under the ROC Curve) treats the Grad-CAM++ heatmap as a probabilistic map and evaluates its ability to distinguish between pixels inside the ground truth lesion mask (true region) and those outside (background). A higher AUC reflects better discriminative capability. In our study, Grad-CAM++ achieved an AUC of 0.9444 for hemorrhagic stroke and 0.7773 for ischemic stroke, indicating strong localization performance, especially for hemorrhagic cases where visual contrast is more prominent.
- Dice Coefficient quantifies the spatial overlap between the binary version of the Grad-CAM++ map and the ground truth mask. Although our Dice scores were modest (0.0869 for ischemia and 0.0420 for hemorrhage), this is consistent with the nature of Grad-CAM++—which is optimized for highlighting key discriminative regions, not full lesion segmentation. As such, a low Dice score does not undermine the interpretability of the method but reflects the sparsity of activation maps.
- Average Activation (AvgActivation) calculates the average intensity of Grad-CAM++ activations within the annotated lesion area. Higher AvgActivation implies stronger model focus on clinically relevant zones. Hemorrhagic stroke regions demonstrated significantly higher attention (0.6084) compared to ischemic ones (0.2984), aligning with qualitative visualizations and the higher AUC observed in hemorrhagic cases.

These findings, in conjunction with the qualitative inspection of Grad-CAM++ overlays (Figure 4 and Figure 5), demonstrate that the deep layers of the MaxViT model are effectively capturing diagnostically meaningful regions. Despite modest Dice scores, the consistently high AUC and AvgActivation values provide strong evidence of the model’s attention alignment with clinical expectations, supporting its reliability for explainability in stroke classification.

## 7. Conclusions

This study focused on the early detection and classification of brain stroke types (ischemic, hemorrhagic, or no stroke) using CT-scan images, which remain widely accessible and cost-effective in clinical settings. The significance of this work lies in its contribution to developing early stroke detection systems, which are crucial due to the severe health, social, and economic consequences of strokes. Throughout this study, we reviewed previous studies on stroke detection and analyzed literature on different models to identify limitations, advantages, and gaps in existing DL techniques. The problem was framed as a multiclass classification task to achieve the research objectives, and the dataset was preprocessed to align with the proposed models.

We implemented and evaluated several advanced transformer-based and Hybrid-based architectures, such as TNT, MaxViT, and ConvNext, while employing ViT as a baseline, to assess their effectiveness in stroke classification. Experimental results based on multiple evaluation metrics confirmed that incorporating transformer-based components into DL models high predictive performance, with significant improvements when combined with advanced data augmentation strategies. The inclusion of Grad-CAM++ provided model interpretability, helping bridge the gap between AI predictions and clinical reasoning. While the findings are promising, it is important to note that the dataset used in this study is sourced exclusively from the Republic of Turkey. As such, future work should consider validating the proposed models on external datasets from different populations and clinical environments to further assess generalizability and robustness. To extend and enhance the scope and impact of this work, future directions include:

- Incorporating more geographically and demographically diverse datasets.
- Deploying real-time stroke detection systems for clinical environments.
- Implementing attention-based XAI techniques, such as attention rollout or attention heatmaps, leveraging the inherent attention mechanisms in transformer-based architectures, to provide more transparent and clinically useful explanations of model predictions.
- Integrating multimodal data, such as vital signs, with CT-scan imaging to boost model performance.

Overall, this study offers valuable contributions toward improving stroke management, improving long-term health conditions, reducing mortality rates, and supporting healthcare professionals in clinical decision-making.

## Figures and Tables

**Table 1:** Key Difference between Hemorrhagic and Ischemic Strokes.

Feature	Hemorrhagic Stroke	Ischemic Stroke
CT Appearance	Hyperdense (bright regions)	Hypodense (dark regions)
Lesion Markers	Blood accumulation, Hematoma	Infarcted tissue, Reduced perfusion
Midline Shift	Common due to mass effect	Less common, Unless large infarct
Possible Locations	Basal ganglia, Cerebellum, Brainstem	Middle cerebral artery territory
Detection	Rapid onset, Easily detected on CT	Often detected later, Subtle signs

**Table 2:** Dataset statistics used in this study for brain stroke CT-scan images.

Dataset Name	Type	Number of CT Scans
CT-scan Images from Republic of Turkey’s Ministry of Health’s [28].	Ischemic Stroke	1130
	Hemorrhagic Stroke	1093
	Normal (No Stroke)	4427
	All classes (Total images)	6650

**Table 3:** Hyperparameter Configuration. Bold indicates the selected values.

Hyperparameter	Values
Optimizer	**Adam**, mAdam
Learning Rate	1 × 10^−3^, 1 × 10^−5^, **3 × 10^−4^**
Batch Size	16, 32, **64**
Number of Epochs	**25**, 40, 50, 100
Drop out ratio	0.03, 0.04, **0.05**

**Table 4:** Prediction performance of transformer-based and hybrid pretrained models for multiclass stroke classification. Models were evaluated under three training settings: original dataset, classical augmentation, and cGAN-based augmentation. Bold font with underline indicates the best results, and bold font alone shows the second-best results.

DL Models	Accuracy	F1-Score	Recall	Precision	AUC
ViT	0.8662	0.8539	0.8541	0.8539	0.9250
ViT + classical DA	0.8556	0.8420	0.8436	0.8410	0.9358
ViT **+ cGAN**	0.8964	0.9084	0.9091	0.9085	0.9753
TNT	0.9286	0.9405	0.9414	0.9411	0.9926
TNT + classical DA	0.9383	0.9238	0.9248	0.9242	0.9911
TNT **+ cGAN**	0.9600	0.9603	0.9606	0.9609	0.9953
ConvNext	0.9617	0.9569	0.9571	0.9570	0.9766
ConvNext + classical DA	0.9564	0.9580	0.9579	0.9589	0.9611
ConvNext **+ cGAN**	0.9745	0.9646	0.9648	0.9651	0.9657
MaxViT	0.9759	0.9683	0.9684	0.9683	**0.9975**
MaxViT + classical DA	**0.9789**	**0.9797**	**0.9797**	**0.9800**	0.9973
MaxViT + **cGAN**	**0.9800**	**0.9799**	**0.9800**	**0.9803**	**0.9956**

**Table 5:** Comparative performance of the proposed transformer-based models against a recent baseline study on stroke classification using CT images. Bold font with underline indicates the best results, and bold font alone shows the second-best results.

Study	Models	Accuracy	F1-Score	Recall	Precision
Baseline Study [28]: Classification of brain ischemic and hemorrhagic stroke Study	EfficientNet-B0 + SVM	0.9513	0.9494	0.9493	0.9506
Proposed Method (three models with data augmentation using cGAN)	MaxViT + cGAN	**0.9800**	**0.9799**	**0.9800**	**0.9803**
	ConvNext + cGAN	**0.9745**	**0.9646**	**0.9648**	**0.9651**
	TNT + cGAN	0.9600	0.9603	0.9606	0.9609

**Table 6:** Quantitative Evaluation of Grad-CAM++ Explanations Across Stroke Types.

Stroke Type	AUC	Dice Coefficient	AvgActivation
Hemorrhagic Stroke	0.7773	0.0869	0.2984
Ischemic Stroke	0.9444	0.0420	0.6084

## Data Availability

The datasets used for this research are available upon request.

## Abbreviations

The following abbreviations are used in this manuscript:

CT	Computed tomography
MRI	Magnetic resonance imaging
AI	Artificial intelligence
XAI	Explainable AI
ML	Machine learning
DL	Deep learning
TL	Transfer learning
SVM	Support vector machine
MLP	Multi-Layer perceptron
LDA	Linear discriminant analysis
SCM	Structural Co- occurrence matrix
CNN	Convolutional neural network
ReLU	Rectified Linear Unit
ViT	Vision transformer
ConvNext	Convolutional neural network next
MaxViT	Multi-Axis vision transformer
TNT	Transformer in transformer
DA	Data augmentation
cGAN	Conditional Generative Adversarial Network
